# Effect of In/Al ratios on structural and optical properties of InAlN films grown on Si(100) by RF-MOMBE

**DOI:** 10.1186/1556-276X-9-204

**Published:** 2014-05-01

**Authors:** Wei-Chun Chen, Yue-Han Wu, Chun-Yen Peng, Chien-Nan Hsiao, Li Chang

**Affiliations:** 1 Instrument Technology Research Center, National Applied Research Laboratories, 20 R&D Rd. VI, Hsinchu Science Park, Hsinchu 30076, Taiwan; 2 Department of Materials Science and Engineering, National Chiao Tung University, 1001, Tahsueh Road, Hsinchu 30010, Taiwan

**Keywords:** InAlN, In/Al ratios, RF-MOMBE

## Abstract

In_*x*_Al_1-*x*_N films were deposited on Si(100) substrate using metal-organic molecular beam epitaxy. We investigated the effect of the trimethylindium/trimethylaluminum (TMIn/TMAl) flow ratios on the structural, morphological, and optical properties of In_*x*_Al_1-*x*_N films. Surface morphologies and microstructure of the In_*x*_Al_1-*x*_N films were measured by atomic force microscopy, scanning electron microscopy, X-ray diffraction (XRD), and transmission electron microscopy (TEM), respectively. Optical properties of all films were evaluated using an ultraviolet/visible/infrared (UV/Vis/IR) reflection spectrophotometer. XRD and TEM results indicated that In_*x*_Al_1-*x*_N films were preferentially oriented in the *c*-axis direction. Besides, the growth rates of In_*x*_Al_1-*x*_N films were measured at around 0.6 μm/h in average. Reflection spectrum shows that the optical absorption of the In_*x*_Al_1-*x*_N films redshifts with an increase in the In composition.

## Background

Recently, InAlN film is a highly attractive III-nitride semiconductor with numerous potential applications because InAlN has band gap energy in the range from 6.2 eV for AlN to 0.7 eV for InN. Therefore, InAlN alloys are attractive for possible applications in light-emitting diode (LEDs) and high-efficiency multijunction tandem solar cell in the wide spectral range from ultraviolet to infrared [1-3]. In addition, compared with Ga(In, Al)N, InAlN has not been so intensively investigated because the growth of InAlN suffers from the difficulty of phase separation due to large immiscibility, optimum growth temperatures, lattice constant, bonding energy, and difference of thermal stability between InN and AlN [4]. Moreover, few studies have been performed because InAlN has an unstable region concerning miscibility [5]. Therefore, it was very difficult to grow high-quality InAlN since there were many variables in the growth condition.

Previous studies of InAlN growth on an AlN buffer layer show that it has improved the crystallinity of the InAlN films and prevented oxygen diffusion from the substrate [6]. Besides, the growth of the InAlN film in all composition regions has been realized with the molecular beam epitaxy (MBE) growth method [7], while it was reported that In-rich InAlN with an In content >32% grown by metal-organic vapor phase epitaxy (MOVPE) showed the phase separation [8]. Also, Houchin et al. indicated that the film quality of InAlN was degraded with increasing Al content. However, phase separation is not observed for the films obtained in their study [9]. Kariya et al. conclude that lattice matching is important in order to grow high-quality InAlN with a smooth surface morphology [10]. Especially, Guo and coworkers [11] fabricated the first single-crystal Al_*x*_In_1-*x*_N films with *x* being from 0 to 0.14 in the low-Al composition regime using MOVPE. On the other hand, Sadler et al. indicated that trimethylindium flux was increased; the indium incorporation initially increased but then leveled off; and for further increases, the amount of indium on the surface as droplets increases significantly [12]. Various growth techniques have been used for growth of InAlN films, such as radio-frequency molecular beam epitaxy (RF-MBE) [13], metal-organic chemical vapor deposition (MOCVD) [14], pulse laser deposition (PLD) [15], and magnetron sputtering [16].

On the other hand, silicon is a very promising material for growth of III-nitride materials, with its good thermal conductivity which is especially interesting for electronic applications [17] and also for low-cost light-emitting diode (LED) applications [18]. Also, very few studies indicated that In-rich InAlN films were grown on Si substrate using radio-frequency metal-organic molecular beam epitaxy (RF-MOMBE), although InAlN films often were grown by MOCVD and MBE methods. Compared with the MOCVD method, the RF-MOMBE technique generally has the advantage of a low growth temperature for obtaining epitaxial nitride films [19,20]. Also, our previous study indicated that the RF-MOMBE growth temperature for InN-related alloys was lower than the MOCVD growth temperature [21].

In this paper, the InAlN films were grown on Si(100) by RF-MOMBE with various trimethylindium/trimethylaluminum (TMIn/TMAl) flow ratios. Structural properties and surface morphology are characterized by high-resolution X-ray diffraction (HRXRD), transmission electron microscopy (TEM), atomic force microscopy (AFM), and scanning electron microscopy (SEM). Optical properties of all InAlN films were also investigated by an ultraviolet/visible/infrared (UV/Vis/IR) reflection spectrophotometer with integrating sphere.

## Methods

**Figure 1:**
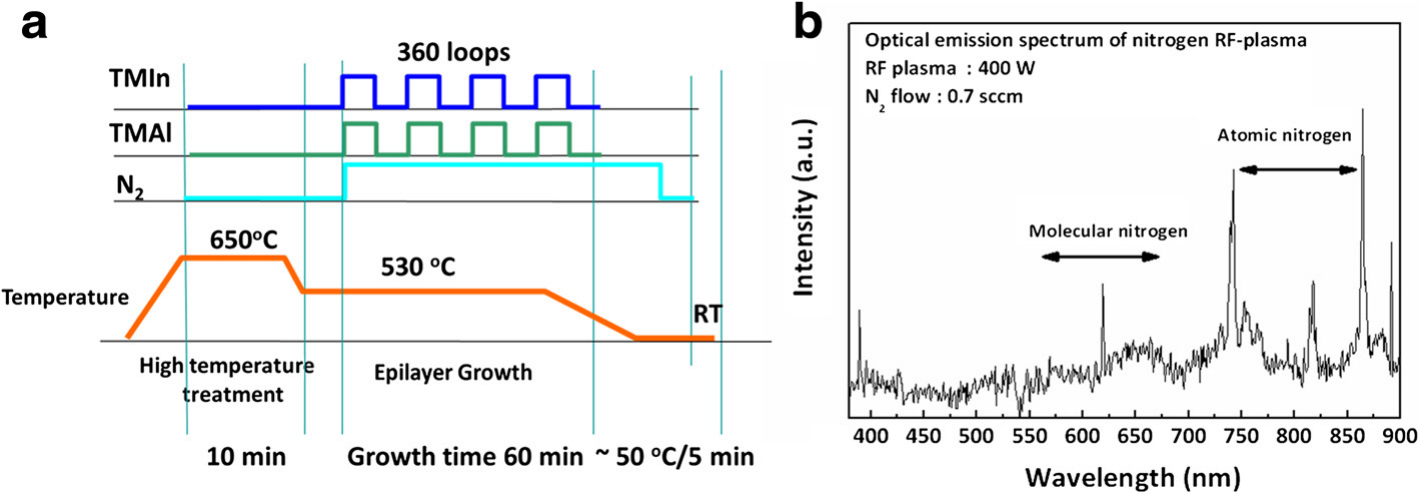
**Growth sequence of RF-MOMBE and spectrum of a nitrogen RF plasma. (a)** Growth sequence of RF-MOMBE pulses for InAlN films. **(b)** A typical optical emission spectrum of a nitrogen RF plasma at 400 W/0.7 sccm.

Highly *c*-axis-oriented InAlN films were deposited on Si(100) substrate using RF-MOMBE. The RF-MOMBE growth chamber was evacuated to a base pressure of 5 × 10^-9^ Torr by a turbomolecular pump. TMIn and TMAl without any carrier gas were used for group III precursor. The active nitrogen radicals were supplied by a radio-frequency plasma source (13.56 MHz). TMAl and TMIn precursors were kept at room temperature and 55°C, respectively. By changing the TMIn/TMAl flow ratio from 1.29 to 1.63 under a constant nitrogen supply with a flow rate of 0.7 sccm and an RF plasma power of 400 W, InAlN films were grown at 530°C for 1 h to investigate the effect of the V/III ratio. The Si(100) substrates were cleaned in a wet bench using Radio Corporation of America (RCA) processes for about 30 min. Also, the substrate followed wet etch in buffered oxide etch (BOE) for 30 s, and then into the growth chamber for InAlN growth. Prior to InAlN growth, the Si substrate in base pressure (5 × 10^-9^ Torr) was heated at 650°C for 10 min for substrate surface cleaning. After, the substrate temperature was decreased to 530°C for all InAlN film growth. During the deposition, the substrate temperature was monitored by a thermocouple (contact with heater backside). The growth sequence of the unit cells of TMIn/TMAl is described in Figure 1a. There are three unit cells; 10-s pulses of TMIn, 10-s pulse of TMAl, and normal open of atomic nitrogen were introduced alternately into the growth chamber. Figure 1b shows the optical emission spectrum of the nitrogen RF plasma with a nitrogen pressure of 7 × 10^-6^ Torr in the growth chamber. It is notable that there are a number of emission peaks associated with molecular and atomic nitrogen transitions that appear in this spectrum. The dominant emission peaks at 740, 820, and 860 nm indicate that a significant amount of atomic nitrogen is produced in the N_2_ RF plasma.

The X-ray diffraction (Siemens D5000, Siemens Co., Munich, Germany) measurements were carried out in a *θ*-2*θ* coupled geometry using Cu-*K*α radiation to identify the presence of secondary phases or crystalline structures. The lattice parameters of In_*x*_Al_1-*x*_N films and the value of *x* were calculated by high-resolution X-ray diffraction (Bruker D8, Bruker Optik GmbH, Ettlingen, Germany). The diffraction angle 2*θ* was scanned from 20° to 40° at 0.005°/s. The surface and cross-sectional morphologies of the In_*x*_Al_1-*x*_N films were analyzed using a field-emission scanning electron microscope (FE-SEM, Hitachi S-4300, Hitachi, Ltd., Chiyoda, Tokyo, Japan). The microstructure of the InAlN films was investigated in detail by TEM in cross-sectional configuration (TEM, Philips Tecnai 20 (FEI/Philips Electron Optics, Eindhoven, Netherlands) and JEOL 2010 F (JEOL Ltd., Akishima, Tokyo, Japan)). The In_*x*_Al_1-*x*_N film's composition was determined with HRXRD. The optical reflectance measurements were performed by using a UV/Vis/IR reflection spectrophotometer with integrating sphere (PerkinElmer Lambda 900, PerkinElmer, Waltham, MA, USA) from 200 to 2,000 nm.

## Results and discussion

**Figure 2:**
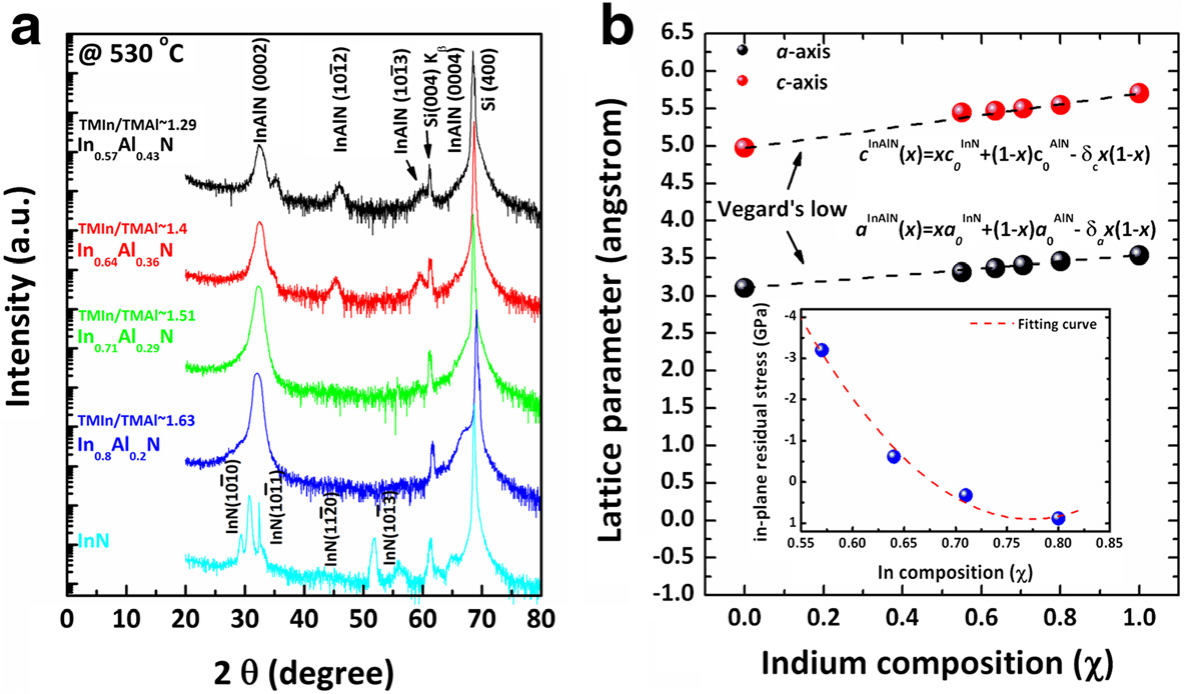
**XRD analysis of InAlN films. (a)** *θ*-2*θ* XRD pattern of InAlN films deposited on Si(100) with various In compositions. **(b)** Composition dependence of the calculated *a*-axis and *c*-axis lattice parameters of InAlN alloys.

Figure 2a shows the *θ*-2*θ* scan XRD pattern for the InAlN films grown at 530°C with the TMIn/TMAl flow ratio of 1.29, 1.4, 1.51, and 1.63. The XRD pattern indicated that the peaks corresponding to InAlN (0002), ( $10\bar{1}1$), ( $10\bar{1}2$), and ( $10\bar{1}3$) were observed for InAlN films grown on the Si(100) substrate. Also, the XRD results of InN and InAlN films reveal that all the films are of wurtzite structure which is preferentially oriented in the *c*-axis direction. No metallic indium peak was detected in the XRD pattern. In addition, it is clearly observed that peaks of all InAlN shifted depending on In composition. However, the crystalline quality of the InAlN films degrades with increasing Al content. The result is in agreement with the report of Houchin et al*.*[9].

Vegard's law [22] has been applied to determine the average In composition of the ternary alloy films via measurement of lattice parameters from HRXRD.

Assuming Vegard's law to hold for In_*x*_Al_1-*x*_N and considering the biaxial strain in the layer, the indium composition can be determined by applying the relation. Therefore, the exact indium mole fraction *x* of the alloy, considering the deformation of the unit cell, is

$$x=-\frac{\text{ac}\left(1+v_{\left(x\right)}\right)-{\text{ac}}_{0}^{\text{AlN}}-a_{0}^{\text{AlN}}cv_{\left(x\right)}}{{\text{ac}}_{0}^{\text{AlN}}-{\text{ac}}_{0}^{\text{InN}}-a_{0}^{\text{InN}}{\text{cv}}_{\left(x\right)}+a_{0}^{\text{AlN}}{\text{cv}}_{\left(x\right)}}$$

where *ν*_(*x*)_ is Poisson's ratio defined as *ν*_(*x*)_ = 2*C*_13_/*C*_33_; *C*_13_ and *C*_33_ are the elastic constants of the hexagonal III-nitrides. The material constants used in this study are *a* = 0.311 nm, *c* = 0.498 nm [23], *C*_13_ = 99 GPa, and *C*_33_ = 389 GPa for AlN [24]; and *a* = 0.354 nm, *c* = 0.5706 nm [23], *C*_13_ = 121 GPa, and *C*_33_ = 182 GPa for InN [25]. For In_*x*_Al_1-*x*_N ternary alloy, both lattice constants and Poisson's ratio *v*(*x*) are obtained by linear interpolation from the values of binaries. As a result, it can be concluded that the molar fraction of InN on a biaxially strained In_*x*_Al_1-*x*_N film is the only possible solution between 0 and 1 for the following third-order equation which presents *x* as a function only of two variables. The In composition (*x*) is accordingly to be calculated as *x* = 0.57 ± 1% (TMIn/TMAl, approximately 1.29), 0.64 ± 1% (TMIn/TMAl, approximately 1.4), 0.71 ± 1% (TMIn/TMAl, approximately 1.51), and 0.80 ± 1% (TMIn/TMAl, approximately 1.63) by Vegard's law.

The XRD pattern of an In content of <0.64 exhibits extremely weak and broad peaks, which indicates that the film is of poor quality due to structural defects. Also, the In_0.64_Al_0.36_ N film shows a polycrystalline structure, suggesting that the in-plane residual stress of the In_0.64_Al_0.36_ N film is almost relaxed after growth.

At above *x* = 0.71, the pattern indicates that the InAlN films are preferentially oriented in the *c*-axis direction. In addition, a weak shoulder peak (2*θ*, approximately 31.909°) was detected at the highest In content of approximately 0.71, indicating an intermediate layer between the film and the Si substrate. As can be seen in Figure 2b, the lattice parameters for *c*-axis and *a*-axis obtained from symmetric (0002) and asymmetric ( $10\bar{1}2$) diffractions of InAlN increased with the increase of In content. The results agree with the theoretical calculations and report of Guo et al. [26].

Figure 2b shows the calculated lattice parameters of all In_*x*_Al_1-*x*_N films with various In compositions. Both *c* and *a* lattice parameters exhibit essentially a linear dependence on the In composition with very small deviations from Vegard's law. In our results, the bowing parameters of δ_*a*_ = 0.0412 ± 0.0039 Å and δ_*c*_ = -0.060 ± 0.010 Å describe the deviations from Vegard's rule. Therefore, the variation of the In_*x*_Al_1-*x*_N lattice parameters with In content *x* can be approximated as follows:

$$\begin{array}{l} c^{\text{InAlN}}\left(x\right)=xc_{0}^{\text{InN}}+\left(1-x\right)c_{0}^{\text{AiN}}-\delta_{c}x\left(1-x\right)\\ a^{\text{InAlN}}\left(x\right)=xa_{0}^{\text{InN}}+\left(1-x\right)a_{0}^{\text{AiN}}-\delta_{a}x\left(1-x\right) \end{array}$$

where InN and AlN lattice parameters are based on a previous study (for InN, *a* = 3.538 Å and *c* = 5.706 Å [27]; for AlN, *a* = 3.11 Å and *c* = 4.98 Å) [23].

The lattice parameter of the In_0.57_Al_0.43_ N film was calculated to be larger than the theoretical value, which may be caused by phase separation and/or lattice strain. The in-plane residual stress of all InAlN films is shown in the inset of Figure 2b. The residual stress was tensile at an In content of >71%. The compressive stresses occurred in the films deposited at an In content of <64%. When the In content is high (>71%), small tensile intrinsic stresses are observed. It has been proposed that one reason for the occurrence of tensile intrinsic stresses is the existence of numerous grain boundaries. Therefore, small tensile residual stresses were obtained at an In content of >71%, and large compressive stresses were obtained at In composition *x* = 0.57.

**Figure 3:**
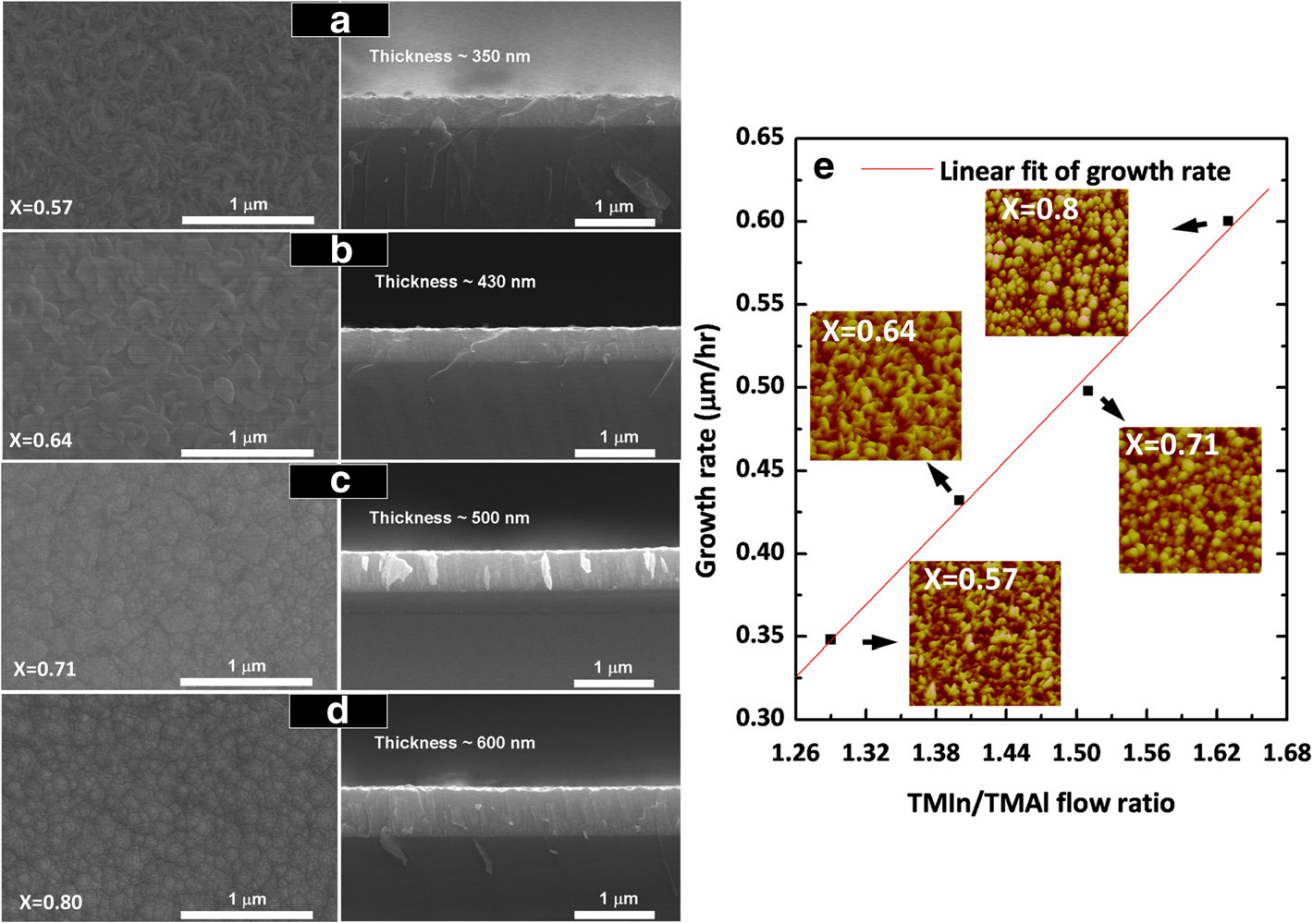
**SEM cross-sectional images. (a-d)** Top-view and cross-sectional SEM images of In_*x*_Al_1-*x*_N films. **(e)** Growth rate of InAlN films with various In compositions.

Figure 3a,b,c,d shows surface morphologies and cross section of In_*x*_Al_1-*x*_N films which were prepared on Si(100) with different In/Al ratios. Also, the surface roughness is larger than in other reports [28] due to high-density grain boundaries and island growth. Besides, the grain size of In_*x*_Al_1-*x*_N decreases with the increase of TMIn mass flow which may be due to the indium interstitials. Thus, both AFM and SEM measurement results show that the use of smaller TMIn mass flow leads to a reduction in the surface roughness of the InAlN film. Also, the thickness of the grown InAlN in this study was increased with increasing TMIn mass flow. Besides, growth rates of all InAlN films were around 0.35 μm/h at *x* = 0.57, 0.43 μm/h at *x* = 0.64, 0.5 μm/h at *x* = 0.71, and 0.6 μm/h at *x* = 0.80, respectively. Moreover, the surface of In_0.80_Al_0.2_ N film was clearly observed to be rough, as compared with those of the other reports of In_*x*_Al_1-*x*_N layers [16]. Figure 3e shows that the growth rate depended on the TMIn mass flow. It is clearly seen that by increasing the TMIn/TMAl flow ratios from 1.29 to 1.63, the growth rate of the films was increased from 0.35 to 0.6 μm/h. However, the increase of the surface roughness with the increase of growth rate may be due to the 3-D growth mode. The insets in Figure 3e show the AFM images corresponding to SEM images of the surface morphologies for the InAlN films.

**Figure 4:**
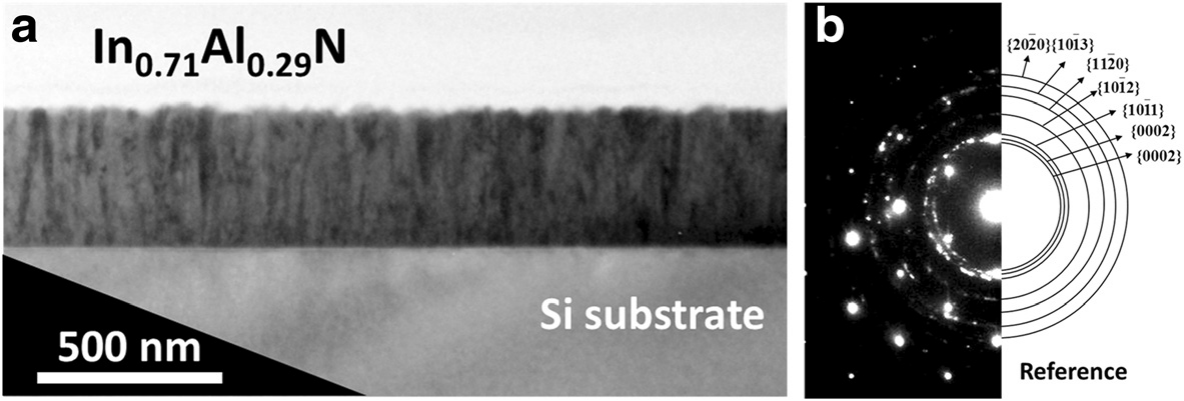
**TEM images of the cross section of In**_**0.71**_**Al**_**0.29**_ **N/Si. (a)** Cross-sectional TEM image and **(b)** the SAD pattern from the In_0.71_Al_0.29_ N film.

Figure 4a shows a cross-sectional bright-field TEM image of the In_0.71_Al_0.29_ N film. The image clearly shows that the structural characteristics of the In_0.71_Al_0.29_ N film exhibited a rough surface and columnar structure at the cleavage. In addition, existence of no metallic In inclusions can be observed in the images which agree with the XRD results. Besides, the selected-area diffraction pattern (SAD) reveals InAlN/Si reflections shown Figure 4b. Individual diffraction rings can be identified as InAlN reflections, indicating that it is a polycrystalline InAlN film with preferred *c*-axis.

**Figure 5:**
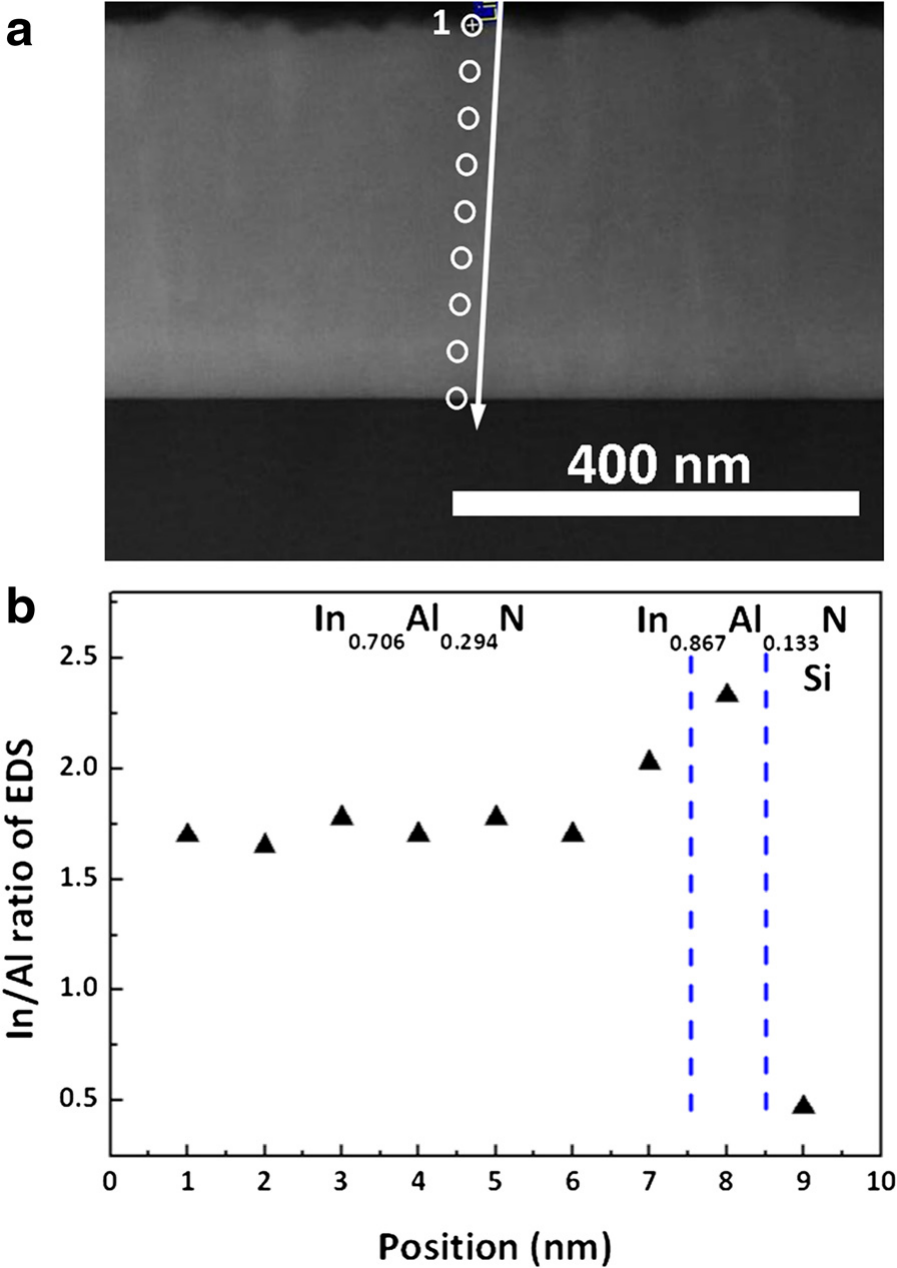
**HAADF analysis of In**_**0.71**_**Al**_**0.29**_ **N films. (a)** HAADF micrograph and **(b)** EDS line scan of the In_0.71_Al_0.29_ N film.

Figure 5a shows the high-angle annular dark-field (HAADF) cross-sectional image of the In_0.71_Al_0.29_ N film which is taken in the [110]_Si_ zone axis projection. The image shows that the two layers are visible. The top layer exhibited a thickness of about 420 nm which was measured at an indium content *x* of approximately 0.71 by scanning transmission electron microscopy with energy-dispersive spectroscopy (STEM-EDS). The bright layer of about 80 nm was observed at bottom regions which are indium-rich. On the other hand, the STEM-EDS line scan profile shows between InAlN films to Si substrate as shown in Figure 5b. From the top layer, cross-sectional line scan profiling of the InAlN film showed that the major In and Al elements were homogeneously distributed over the cross section of the stem. The result was observed to be similar to MOCVD growth of AlInN films on the GaN layer [29]. The average concentrations in the brighter regions are roughly estimated to be 70% ± 5% In and 30% ± 5% Al, while the concentrations in the darker areas are 64% ± 5% In and 36% ± 5% Al.

**Figure 6:**
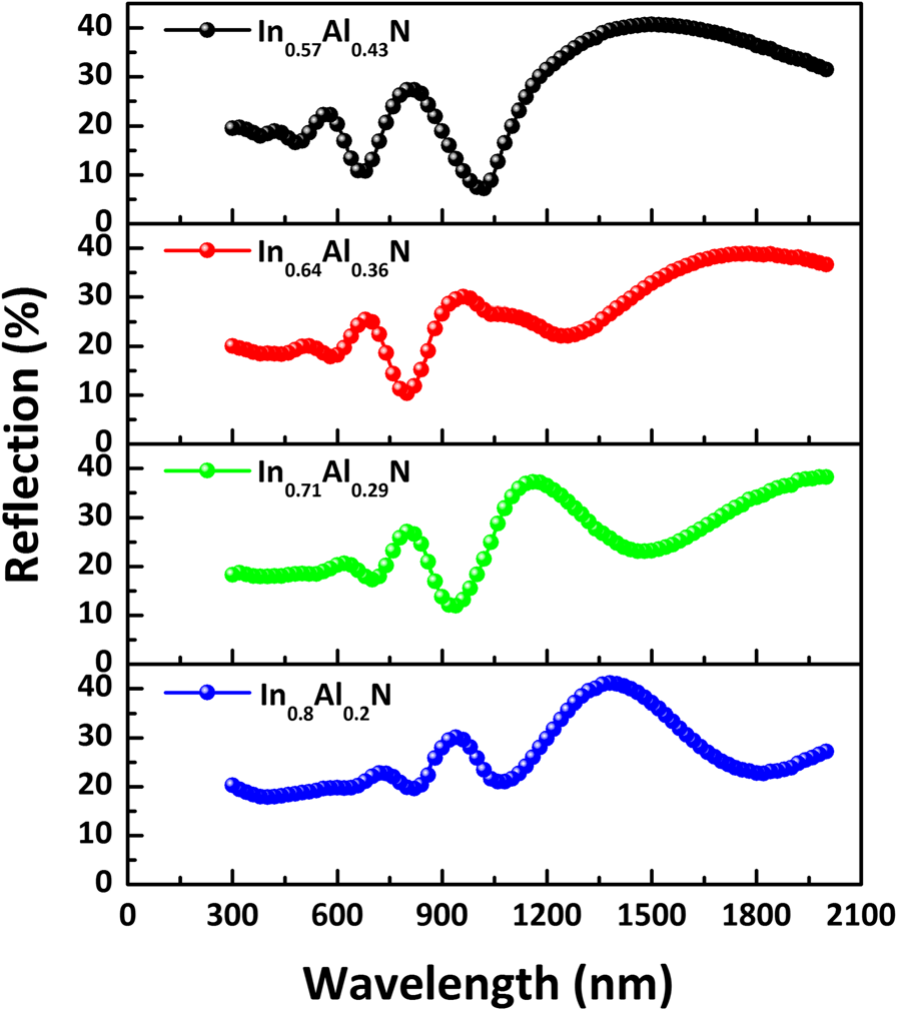
**Reflection spectra of In**_***x***_**Al**_**1-** ***x***_**N films at various in compositions.**

The optical properties of In_*x*_Al_1-*x*_N films were investigated by measuring the optical reflectance spectra on a UV/Vis/IR spectrophotometer with integrating sphere (200 to 2,000 nm). The reflectance spectra of all In_*x*_Al_1-*x*_N films are as shown in Figure 6. Prominent Fabry-Perot interference fringes attributed to multiple-layer-substrate reflections are observed at a long wavelength. However, Fabry-Perot interference fringes increase with the increase of film thickness, since the interference fringe begins to disappear in the vicinity of the wavelength related to the optical absorption edge [30]. In addition, light scattering-induced changes may have occurred in the surface of polycrystalline InAlN films due to surface roughness such as grain, grain boundaries, and microscopic pores. The reflection spectra shows that the optical absorption of the InAlN films redshifts with an increase in the In composition *x*.

## Conclusions

Highly *c*-axis-oriented In_*x*_Al_1-*x*_N films were grown on Si(100) by RF-MOMBE. From XRD results, In_0.71_Al_0.29_ N has the best crystalline quality among the In_*x*_Al_1-*x*_N samples for various values of the In composition fraction *x* studied here. However, the strain of all InAlN films has not been relaxed after growth. At an In content of <57%, the InAlN/Si(100) exhibits worse crystallinity which observed obviously large residual stress. The surface roughness of InAlN films increased with the increase of In composition. The corresponding reflection spectra of the In_*x*_Al_1-*x*_N films are observed at around 1.5 to 2.55 eV.

## Competing interests

The authors declare that they have no competing interests.

## Authors’ contributions

WCC designed and carried out the experiment and statistical analysis, and participated in the drafting of the manuscript. YHW helped with the transmission electron microscopy experiments. CYP carried out the high-resolution X-ray measurements. CNH revised the manuscript. LC was involved in the discussions of experimental results. All authors read and approved the final manuscript.
